# Brain transcriptome analysis reveals novel long non-coding RNAs in *Dryophytes arenicolor* (Canyon Treefrog)

**DOI:** 10.1093/g3journal/jkaf283

**Published:** 2025-11-22

**Authors:** Héctor Herrera-Orozco, Carolina Rodríguez-Ibarra, Cristian Ivan Hernández-Herrera, Clara Estela Díaz-Velásquez, Felipe Vaca-Paniagua, Hibraim Adán Pérez-Mendoza

**Affiliations:** Posgrado en Ciencias Biológicas, Universidad Nacional Autónoma de México, Ciudad Universitaria, Coyoacán, CDMX 04510, México; Facultad de Estudios Superiores Iztacala, Laboratorio de Ecología Evolutiva y Conservación de Anfibios y Reptiles, Universidad Nacional Autónoma de México, Tlalnepantla de Baz 54090, México; Facultad de Estudios Superiores Iztacala, Unidad de Investigación en Biomedicina, Universidad Nacional Autónoma de México, Tlalnepantla de Baz 54090, México; Posgrado en Ciencias Biológicas, Universidad Nacional Autónoma de México, Ciudad Universitaria, Coyoacán, CDMX 04510, México; Facultad de Estudios Superiores Iztacala, Laboratorio de Ecología Evolutiva y Conservación de Anfibios y Reptiles, Universidad Nacional Autónoma de México, Tlalnepantla de Baz 54090, México; Facultad de Estudios Superiores Iztacala, Unidad de Investigación en Biomedicina, Universidad Nacional Autónoma de México, Tlalnepantla de Baz 54090, México; Facultad de Estudios Superiores Iztacala, Laboratorio Nacional en Salud: Diagnóstico Molecular y Efecto Ambiental en Enfermedades Crónico-Degenerativas, Universidad Nacional Autónoma de México, Tlalnepantla de Baz 54090, México; Facultad de Estudios Superiores Iztacala, Unidad de Investigación en Biomedicina, Universidad Nacional Autónoma de México, Tlalnepantla de Baz 54090, México; Facultad de Estudios Superiores Iztacala, Laboratorio Nacional en Salud: Diagnóstico Molecular y Efecto Ambiental en Enfermedades Crónico-Degenerativas, Universidad Nacional Autónoma de México, Tlalnepantla de Baz 54090, México; Facultad de Estudios Superiores Iztacala, Laboratorio de Ecología Evolutiva y Conservación de Anfibios y Reptiles, Universidad Nacional Autónoma de México, Tlalnepantla de Baz 54090, México

**Keywords:** anuran, lncRNAs, transcriptomics, gene regulation, *Dryophytes arenicolor*

## Abstract

Anurans possess complex genomes, making molecular research challenging, particularly in non-model species. Transcriptomics offers a powerful tool for uncovering genomic responses to environmental changes through distinct transcriptional patterns. A sizeable portion of the transcriptome consists of non-coding RNAs, with long non-coding RNAs (lncRNAs) playing key roles in gene regulation. Here, we performed de novo transcriptome assembly from brain samples of *Dryophytes arenicolor* across 3 life stages: Pre-metamorphic, Metamorphic Climax, and Adults. By aligning our transcriptomes to LNCipedia, we identified 4,557 previously annotated lncRNAs with potential roles in gene regulation, macromolecule biosynthesis, and chromatin organization. To detect novel lncRNAs, we implemented a bioinformatic pipeline to filter out known mRNAs, small ncRNAs, sequences with coding potential, conserved protein domains, and previously identified *D. arenicolor* mRNAs, identifying 4,836 putative novel lncRNAs. To explore their functional roles, we performed Weighted Gene Correlation Analysis and gene enrichment analysis, revealing that these lncRNAs may be involved in protein heterodimerization, nucleosome assembly, and post-transcriptional regulation of gene expression. To our knowledge, this is the first study to characterize lncRNAs across multiple life stages in *D. arenicolor*, highlighting their potential regulatory functions

## Introduction

Amphibians have the greatest variation in genome size among terrestrial tetrapods, with some salamanders (*Necturus*) reaching over 100 Gb, whereas anurans, such as *Platyplectrum ornatum*, possess genomes as small as ∼1 Gb (Lamichhaney et al. 2021). Among anurans, the average genome size is estimated at around 3 Gb, though this is based on a limited number of sequenced species and can reach up to 10 to 12 Gb in the genus *Bombina* (Kosch et al. 2024; Gregory 2025). Such differences in size and the high percentage of repeated content (up to 82%) make their assembly, annotation, and analysis challenging, particularly for non-model species (Kosch et al. 2025).

For organisms with large, complex genomes, transcriptomics has become a *go-to* tool, striking a balance between cost-effectiveness and the ability to capture dynamic genomic responses (Birol et al. 2015; Ceschin et al. 2020). RNAseq has revealed intricate expression profiles for diverse biological contexts like sex development in *Hoplobatrachus rugulosus* (Tang et al. 2021), resistance to body freezing during winter in *Dryophytes chrysoscelis* (do Amaral et al. 2020), identification of antimicrobial peptides of *Boana pugnax* (Liscano Martinez et al. 2020) and even the dispersal behavior of *Hyla sarda* (Libro et al. 2022). Although anuran transcriptomics is an active research field, little is known about the non-coding side of their transcriptional programs.

ENCODE data suggests that around 80% of the genome is actively transcribed into non-coding RNAs (Djebali et al. 2012; Stamatoyannopoulos et al. 2012). Non-coding RNAs (ncRNAs) include a diverse group of transcripts that are not translated into proteins and represent a higher level of gene regulation, as ncRNAs may interact with multiple effectors downstream (Toden et al. 2021). Based on their function, they are broadly divided into housekeeping ncRNAs—such as rRNAs and tRNAs involved in constitutive processes like protein synthesis or RNA modification (Zhang et al. 2022)—and regulatory ncRNAs, which modulate gene expression and are implicated in multiple biological processes (Nemeth et al. 2024) and diseases, including cancer (Yan and Bu 2021).

Regulatory ncRNAs can be further subdivided by size into small ncRNAs (<200 nt) and long ncRNAs (>200 nt). Long ncRNAs (lncRNAs) resemble mRNAs structurally, they are transcribed by RNA polymerase II, capped, and often polyadenylated and spliced (Choudhuri 2023). LncRNAs function as key regulators of multiple biological processes such as nuclear organization, chromatin remodeling, transcriptional regulation, splicing, and mRNA stability (Jafari-Raddani et al. 2022).

The lncRNA repertoire of anurans spans orders of magnitude, from ∼6,000 loci (Hammond et al. 2017) to >50,000 transcripts in *Rhinella arenarum* (Ceschin et al. 2020) and *Pelobates cultriples* (Liedtke et al. 2022). Although in anurans it has been shown that lncRNAs are highly involved in processes such as embryonic development (Forouzmand et al. 2017), exhibit deep conservation (Weghorst et al. 2024) and alterations to their expression patterns lead to disrupted signaling pathways (Sai et al. 2019; Qi et al. 2021), little is known about the role of lncRNAs in other stages of development during their complex life cycles; therefore, we hypothesize that they could be involved in multiple biological processes throughout development such as cell structure organization, transcription regulation, or metabolism. In this work, we aim to identify both previously annotated and putative novel lncRNAs in various stages of the life cycle of *Dryophytes arenicolor* and evaluate their biological roles.

## Methods

### Study species

*D. arenicolor* is a treefrog 32 to 57 mm (Snout-Vent Length) with a wide distribution that ranges from southern Utah in the United States to the northern regions of Oaxaca, Mexico (Croter 2005). The species is known to inhabit varying climates along its distribution, including pine and deciduous forests to deserts (Hernández-Herrera et al. 2024). The species presents morphological variations such as a rough skin that allows it to resist desiccation (Preest et al. 1992) and their tadpoles are resistant to elevated temperatures—up to 32 °C—(Zweifel 1926–1968). Their wide distribution and varied phenotypes make this organism a great model to evaluate gene expression patterns, as broad distributions have been correlated with species’ capacity to respond to different environmental stressors (Eriksson and Rafajlović 2022); ie phenotypic plasticity.

### Study area

Fieldwork was conducted along the intermittent stream located in the Tepotzotlán Sierra (19°46′36.4″N, 99°15′7.6″W) in northern Estado de México. The climate is Temperate Sub-Humid (Cw0) with a summer rain season (García 2004). Altitude ranges between 2,350 and 2,980 ASL. Local flora is formed of grasslands and xeric scrubs, with remnants of oak forests (Hernández-Herrera and Pérez-Mendoza 2021).

### Biological material

To shed light on the regulatory roles of lncRNAs in our study group, we selected 3 life cycle stages (Gosner 1960) as comparison points for the expression patterns of lncRNAs: (i) Pre-metamorphic (G25; Pre), (ii) Metamorphic Climax (G42, Climax), and (iii) Adults as the final point for the comparison (Supplementary Fig. 1). The Pre and Climax stages were selected due to the different transcriptional programs reported previously in tail reabsorption (Wang et al. 2019) and brain development (Raj et al. 2023), and the Adult stage, thanks to being recognized as the final stage of the anuran life cycle.

### Tissue extraction and RNA purification

To eliminate any potential bias associated with individual samples we took 3 samples per stage—9 samples total—, each sample consisted of 3 different organisms randomly selected (pools). The organisms were submerged in a lidocaine solution (10%) to induce a cardiac arrest. Once organisms were euthanized, we obtained brain tissue for individuals of each life cycle stage and immediately preserved it in RNAlater (ThermoFisher) at −80 °C to stabilize the RNA and avoid degradation.

We put the brains of the 3 individuals on a sterile Petri dish and homogenized them using a sterile scalpel and transferred the lysate to the column of the RNAeasy Micro Kit (Qiagen); we isolated the RNA following the manufacturer's instructions. We quantified total RNA concentration using an N80 NanoPhotometer (Implen) and evaluated RNA integrity with 0.8% agarose gels (80 V, 40 min).

### Library preparation and sequencing

NOVOGENE performed quality control on all samples including re-quantification, agarose integrity gels (Supplementary Fig. 2), and electropherograms to estimate RNA Integrity Numbers (Supplementary Fig. 3) and detect potential contaminants in the samples. The sequencing protocol consisted of (i) rRNA depletion, (ii) RNA fragmentation by enzymatic digestion, (iii) reverse transcription, (iv) forward library construction, (v) dUTP protocol to obtain reverse strand library, (vi) end repair and adaptor ligation, and (vii) Illumina Paired-End 150 bp sequencing using a NovaSeq 6000 sequencer.

### Sequencing quality control

To evaluate the quality of the sequencing step, we used FastQC-v0.12.1 (Babraham Bioinformatics 2019) and Trimmomatic-v0.39 (Bolger et al. 2014) to eliminate adapters, low-quality bases, and unpaired reads. The filtering criteria were Phred >25 by nucleotide, mean Phred >25 in a sliding window of 4 nt mean Phred >25, and a final minimal length of 40 nt. After the cleaning step, we used FastQC to generate the quality graphs per sample and MultiQC-v1.25.1 (Ewels et al. 2016) to merge them into 1 graph.

### De novo transcriptome assembly

We assembled the clean reads into a transcriptome per sample using Trinity-v2.15.1 (Haas et al. 2013). We concatenated all the individual transcriptomes per stage to create a reference transcriptome and then concatenated all the reference transcriptomes to obtain a global reference transcriptome. To remove redundancies and maintain all unique transcripts in our reference transcriptomes we used CD-HIT-v4.8.1 (Fu et al. 2012) with a 95% identity threshold to merge sequences, minimizing information loss.

### Transcriptome quality control

**Fig. 1.**
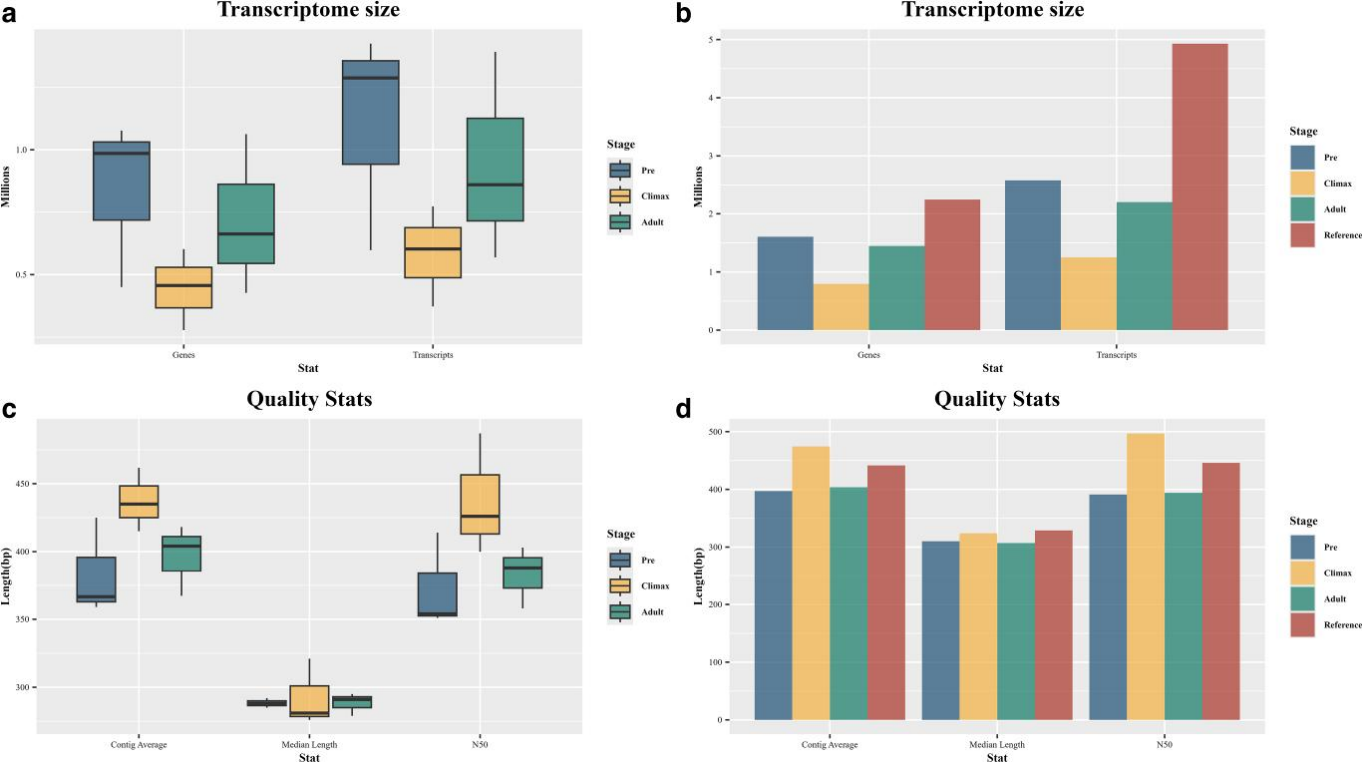
De novo transcriptome size and quality statistics. a) Boxplot of per-sample transcriptome sizes by genes and transcripts. No significant differences were found between stages by gene (*F* = 1.488, *P* = 0.299) or transcripts (*F* = 1.544, *P* = 0.286). b) Bar plot of reference transcriptome sizes by genes and transcripts. The Climax stage showed the smallest reference transcriptome both by genes (700 K) and transcripts (1.5 M). c) Boxplots of quality stats of individual transcriptomes by mean contig length, median contig length, and N50. No significant differences were found between stages by mean contig length (*F* = 2.785, *P* = 0.139), median (*F* = 0.0082, *P* = 0.923), or N50 (*F* = 2.883, *P* = 0.133). d) Bar plot of quality stats of reference transcriptomes. The Climax stage had the lowest mean and median contig length and N50.

To assess the quality of both the individual transcriptomes and the reference assemblies, we used the Trinity auxiliary toolset to quantify a variety of assembly statistics. These included transcriptome size, N50, average and median contig lengths, and Ex90N50 values. To evaluate how well the original reads were represented in each assembled transcriptome, we aligned the raw reads back to their respective de novo assemblies using Bowtie2 v2.5.4 (Langmead and Salzberg 2012). Additionally, we assessed transcriptome completeness by quantifying the presence of Benchmark Universal Single-Copy Orthologs (BUSCOs) using BUSCO v5.8.1 (Manni et al. 2021) with the Eukaryota_odb10, Vertebrata_odb10 and Tetrapoda_odb 10lineage datasets. For statistical analyses, we used the CAR package v-3.1.3 (Fox and Weisberg 2018) to check homogeneity of variances using a Levene test and found that assumptions were met (Fig. 1a, genes: *F* = 0.2396, *P* = 0.7941; transcripts: *F* = 0.2755, *P* = 0.7863; Fig. 1c, contig average: *F* = 0.0661, *P* = 0.9368; median length: *F* = 0.7632, *P* = 0.5066; N50: *F* = 0.1926, *P* = 0.8267) and we used R-v4.3.1 (R Core Team 2023 ) to perform 1-factor ANOVAs and the Tidyverse-v2.0.0 package (Wickham et al. 2019) for data wrangling and visualization.

### Known lncRNAs annotation

To annotate known lncRNAs, we aligned our transcriptomes to the LNCipedia-v5.2 dataset (Volders et al. 2019) using MMSeqs2-v15-6f452 + ds-2 (Steinegger and Söding 2017). Alignments were filtered to retain only hits with >80% (Deng et al. 2018) identity and an *E*-value <0.001, as previously reported (Weisman et al. 2020). We used eulerr package-v7.0.4 (Larsson and Gustafsson 2018) to generate a Venn diagram showing the overlap of annotated lncRNAs among our reference transcriptomes. To investigate the biological roles of these known lncRNAs, we conducted a functional enrichment analysis using g:Profiler2-v0.2.3 (Kolberg et al. 2020). Results with a false discovery rate (FDR) < 0.05 were considered significant.

### De novo lncRNA identification

To annotate de novo lncRNAs we followed the methodology described previously (Kashyap et al. 2020; Motheramgari et al. 2020). We followed a similar methodology as described above to align our transcriptomes and removed protein-coding genes using UniProt database (release October 2024), small non-codingRNAs (sncRNAs), and the list of known lncRNAs identified above (identity >80%, *e*-value <0.001).

To eliminate reads with coding potential we used CPAT-v3.0.0 (Wang et al. 2013). Using the CPAT toolbox we constructed a hexamer frequency table and a logistic regression model with coding probability as the response variable. Both parameters were assembled with coding and non-coding sequences obtained from the *Xenopus laevis* v-10.1 genomic assembly (GCF_017654675.1, [International Xenopus Sequencing Consortium 2021, April 12]). With the statsmodels-v0.14.4 package (Perktold et al. 2010) we used the coding probabilities to calculate an FDR and filter those entries with an FDR < 0.0001.

To eliminate potentially conserved protein domains, we translated the RNA sequences using the EMBOSS-v6.5.7 (Rice et al. 2000) package and aligned them to the Pfam-v38 (Mistry et al. 2021) repository using Hmmer-v3.4 (Eddy 2011) eliminating significant hits (*e*-value < 0.01).

We then aligned our sequences to a protein-coding reference transcriptome of *D. arenicolor* brain constructed in our workgroup (Hernández-Herrera and Pérez-Mendoza, in preparation, available at TSA under BioProject number PRJNA1356325), using MMseqs2 as explained previously. We eliminated all sequences with >95% identity and *e*-value < 0.01.

### WGCNA

To identify potential biological roles of our de novo lncRNAs we first obtained a transcripts per million (TPM)-normalized global expression matrix across our samples using Salmon-v10.0.1 (Patro et al. 2017 ). We eliminated genes with low expression across all samples (*µ* > 1) and selected the topmost variable genes (top 75% of genes by variance).

We fed the expression matrix to the WGCNA-v1.73 (Langfelder and Horvath 2008) to identify potential expression modules. We assigned colors to the matrix and plotted the cluster dendrogram of highly correlated gene modules. For subsequent analyses, we selected only the modules that included any of our de novo lncRNAs. We took the UniProt identifier of the co-expressed protein-coding genes and fed them to the BioMart-v2.58.2 (Smedley et al. 2009) to obtain their respective ENTREZ IDs.

Finally, we used the lists of ENTREZ IDs of each module with clusterProfiler-v4.10.1 (Yu et al. 2012) to obtain the known functions of said proteins in Gene Ontology. We used a Bonferroni correction and considered results with an adj. *P*-value <0.01 as significant. For a comprehensive description of the bioinformatic workflow see Supplementary Fig. 4.

## Results

### Sequencing and transcriptome assembly

Following quality trimming and filtering (Supplementary Figs. 5 and 6), a total of approximately 412 million clean reads were retained, representing 87.1% of the original dataset. GC percentages across samples were around 45%, with curves that resemble a normal distribution. Mean quality scores by sample and by read exceeded Phred 35 in all reads. Adapter sequences, low-quality regions, and uncalled bases were effectively removed. Duplication and overrepresentation levels were comparable to those observed in the raw data.

Using the clean reads, we performed de novo transcriptome assembly for each pool, generating stage-specific reference assemblies and a global reference transcriptome for the complete dataset (Fig. 1a). In the individual assemblies no statistically significant differences were found among stages in number of genes (*F* = 1.488, *P* = 0.299) or transcripts (*F* = 1.554, *P* = 0.286). This trend persisted in the stage-specific reference transcriptomes (Fig. 1b), where the Climax stage showed the fewest genes (∼700 K) and transcripts (∼1.25 M), followed by the Adult stage with ∼1.4 M genes (∼2.2 M transcripts) and finally the Pre-metamorphic stage with around 1.6 M genes and ∼2.5 M transcripts. The global reference transcriptome had ∼2.2 M unique genes and close to 5 M total transcripts.

We evaluated the fragmentation levels of the individual transcriptomes (Fig. 1c) and reference transcriptomes (Fig. 1d). We tested for differences among the individual transcriptomes in mean contig size (*F* = 2.785, *P* = 0.139), contig median (*F* = 0.082, *P* = 0.923), and N50 (*F* = 2.883, *P* = 0.133) and found no statistically significant results. For the reference transcriptomes we found that the Climax stage had the highest contig mean (474.38 bp), median (324 bp), and N50 (497 bp); the Adult and Pre stages showed very similar values, and the Reference transcriptome had a mean contig size of 446 bp, median contig size of 329 bp and N50 of 441.25 bp.

We measured the percentage of representation of the original reads in the assembled transcriptomes (Supplementary Fig. 7). All samples had at least 75% read representation in the final transcriptomes, with the lowest 2 values being the P1 and A1 samples, both with 75.62%; the rest of the samples showed representation levels of ∼90%. We tested for differences in the levels of read representation and found no significant result between stages (*F* = 1.12, *P* = 0.386).

We evaluated the Ex90N50 values (Supplementary Fig. 8) to assess transcript contiguity among highly expressed transcripts. The Climax, Adult, and global reference transcriptomes exhibited identical Ex90N50 curves. In contrast, the curve for the Pre-metamorphic stage showed greater variability among lowly expressed contigs. However, starting at the 40th percentile of expressed transcripts, its curve closely aligned with those of the other stages.

We assessed the completeness of our de novo transcriptomes using the Tetrapoda BUSCO dataset (Table 1). All transcriptomes showed high completeness, with over 80% of BUSCO genes detected as complete. The global Reference transcriptome had the highest completeness at 86.7%. In the stage-specific assemblies, we observed consistently high levels of single-copy genes (∼36%) and duplicated genes (∼30%), while the proportions of fragmented (<7%) and missing (∼13%) BUSCOs were low, with the global reference exhibiting the fewest missing genes.

**Table 1.** BUSCO representation for de novo transcriptomes and the global reference transcriptome across Eukaryota, Vertebrata, and Tetrapoda datasets.

BUSCO	Stage	Complete	Single	Duplicated	Fragmented	Missing
Eukaryota (*N* = 255)	Pre	254 (99.6%)	109 (42.7)	145 (56.9%)	1 (0.4%)	0
	Climax	253 (99.2%)	127 (49.8%)	126 (49.4%)	1 (0.4%)	1 (0.4%)
	Adult	253 (99.2%)	127 (49.8%)	126 (49.4%)	1 (0.4%)	1 (0.4%)
	Reference	255 (100%)	39 (15.3%)	216 (84.7%)	0	0
Vertebrata (*N* = 3354)	Pre	2,916 (87%)	1,240 (37%)	1,676 (50%)	236 (7%)	202 (6%)
	Climax	2,959 (88.2%)	1,402 (41.8%)	1,557 (46.4%)	184 (5.5%)	211 (6.3%)
	Adult	2,920 (87.1%)	1,251 (37.3%)	1,669 (49.8%)	216 (6.4%)	218 (6.5%)
	Reference	3,076 (91.7%)	562 (16.8%)	2,514 (75%)	142 (4.2%)	136 (4.1%)
Tetrapoda (*N* = 5310)	Pre	4,276 (80.5%)	1,860 (35%)	2,416 (45.5%)	355 (6.7%)	679 (12.8%)
	Climax	4,327 (81.5%)	2,011 (37.9%)	2,316 (43.6%)	289 (5.4%)	694 (13.1%)
	Adult	4,248 (80%)	1,836 (34.6%)	2,412 (45.4%)	336 (6.3%)	726 (13.7%)
	Reference	4,603 (86.7%)	936 (17.6%)	3,667 (69.1%)	223 (4.2%)	484 (9.1%)

The table shows stage-specific representation of Complete, Single-Copy, Duplicated, Fragmented, and Missing BUSCO genes, reported as both total counts and percentages.

When assessed with the Vertebrata BUSCO dataset, completeness scores increased across all assemblies, with complete BUSCOs around 90%, reaching 91.7% in the global Reference. These results also reflected higher single-copy gene representation (∼40%) and consistently low levels of fragmented and missing genes (<7%). Using the Eukaryota dataset, all transcriptomes showed nearly complete BUSCO recovery, with over 99% completeness (100% in the Reference transcriptome).

### Detection and distribution of annotated lncRNAs

**Fig. 2.**
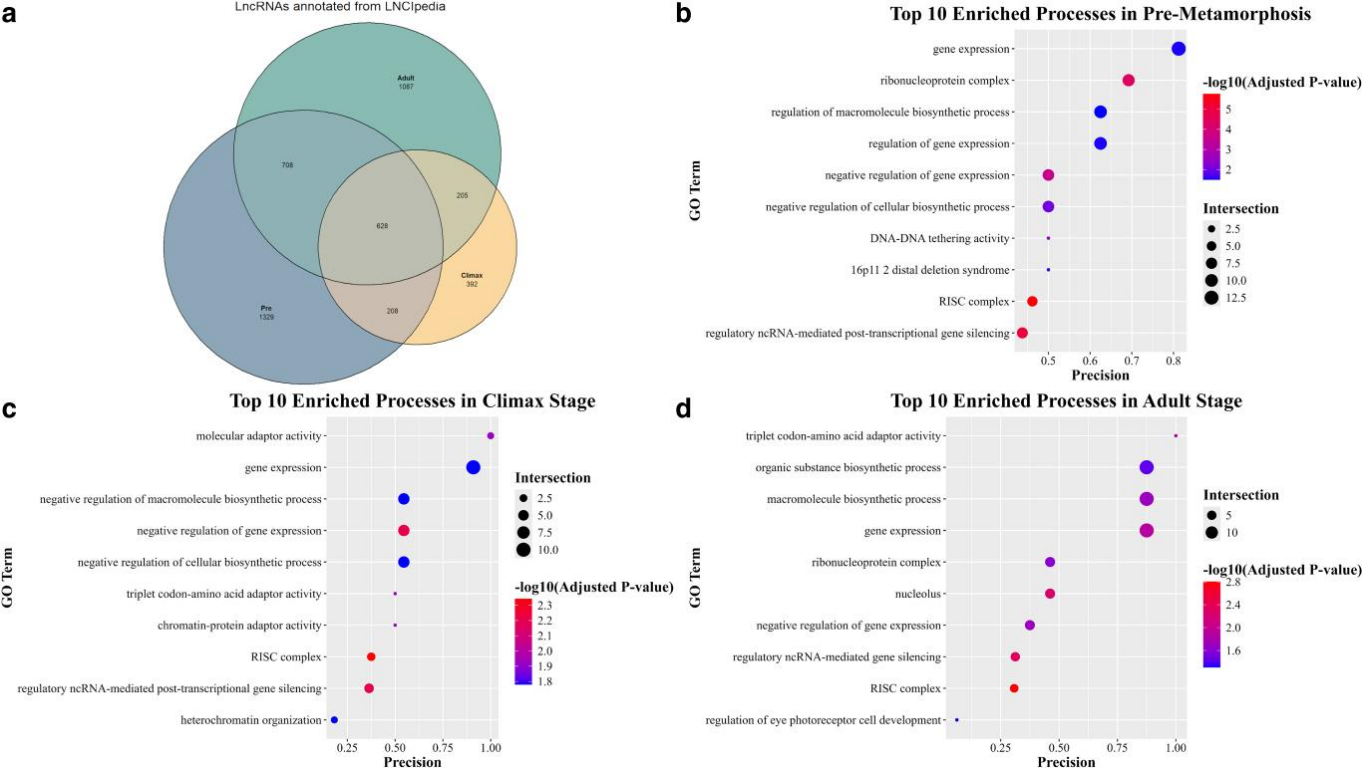
Annotated lncRNA identification in reference transcriptomes. a) Venn diagram of detected known lncRNAs in our dataset. We found 1,329 unique lncRNAs in the Pre-stage, 392 lncRNAs for the Climax stage, and 1,087 genes for the Adult stage. Among the stages, we found 208 common lncRNAs between the Pre and Climax stages, 205 between the Climax and Adult stages, 708 between the Pre and Adult stages, and 628 across all stages. b) Gene enrichment analysis of lncRNAs in the Pre-metamorphic stage, we found significantly enriched processes such as macromolecule synthesis, metabolic involvement, and gene silencing. c) Gene enrichment analysis of lncRNAs in the Climax stage, we found significantly enriched post-transcriptional processes, and heterochromatin formation. d) Gene enrichment analysis of lncRNAs in Adult stages, we detected significantly enriched processes associated with ribonucleoprotein complexes and gene silencing mediated by the RISC complex.

Using LNCipedia, we identified a total of 4,557 known lncRNAs across all de novo assemblies (Fig. 2a). Of these, 1,329 were unique to the Pre-metamorphic stage, 392 lncRNAs were exclusive to the Climax stage, and 1,087 lncRNAs were specific to the Adult stage. Additionally, 628 lncRNAs were common among all 3 stages, while 708 were shared between Pre and Adult, 208 between Pre and Climax stages, and 205 shared genes between Climax and Adult.

We used g:Profiler to search for the biological roles where our lncRNAs could be involved. For the Pre-stage (Fig. 2b), we found lncRNAs associated with relevant biological roles, such as macromolecule synthesis, metabolic roles, and gene silencing. In the Climax stage (Fig. 2c), we obtained genes that regulate biosynthesis, post-transcriptional regulation of gene expression, heterochromatin formation, and processes associated with sexual differentiation. The Adult stage (Fig. 2d) showed enriched processes such as signal recognition, ribonucleoprotein complexes, and gene expression regulation via the RNA-induced silencing complex (RISC).

### Discovery of novel lncRNAs

**Fig. 3.**
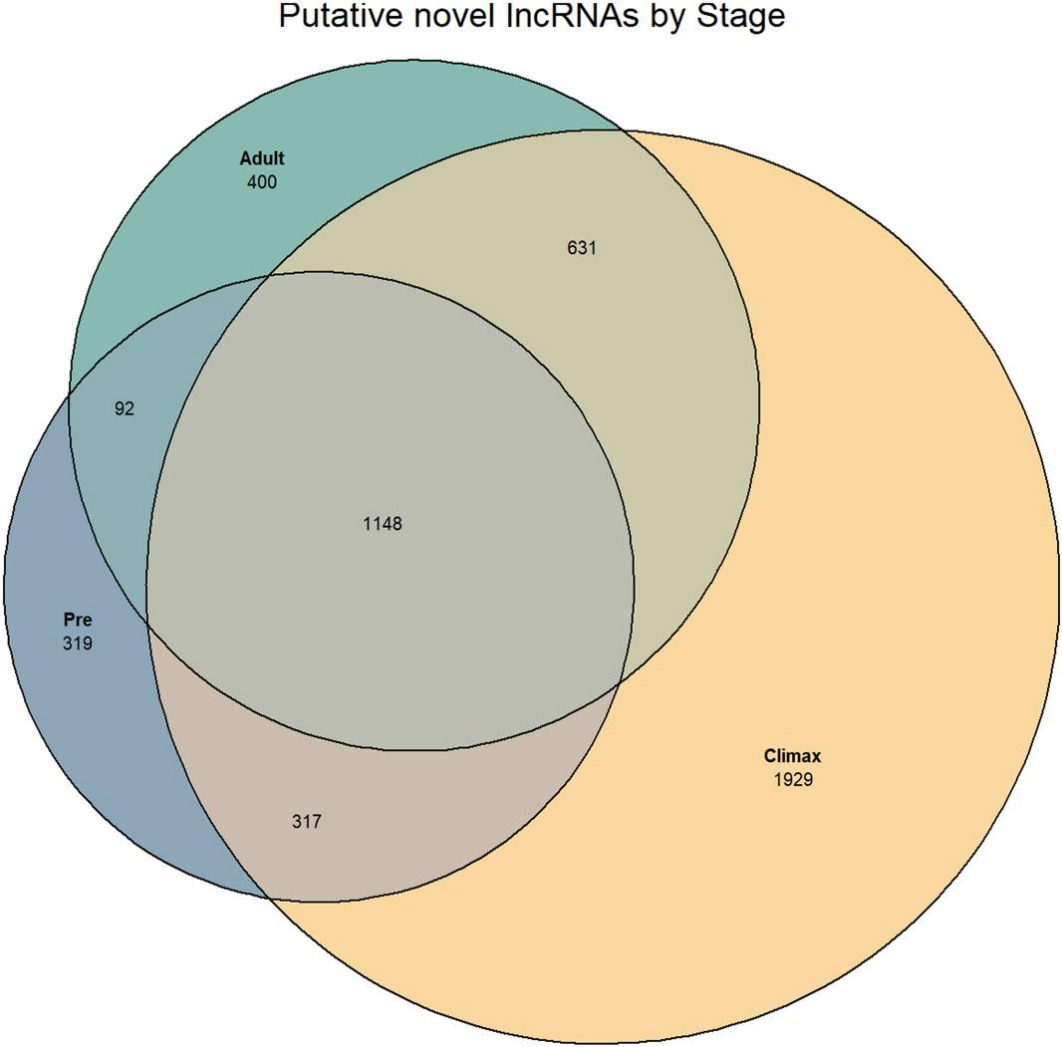
Putative lncRNAs identified de novo Venn diagram of the unique and shared putative lncRNAs shared among stages. We found 319 unique lncRNAs in the Pre-metamorphic stage, 1,929 in the Climax stage, and 400 in the Adult stage. Shared among our groups, we found 317 between the Pre and Climax stages, 631 between the Climax and Adult stages, and 92 between the Pre and Adult stages. Among all stages, we found 1,148 common lncRNAs.

After sequential filtering (Supplementary Fig. 9), we identified 4,836 putative de novo lncRNAs (Fig. 3). Of these, 319 genes were unique to the Pre-metamorphic stage, 1,929 were specific to the Climax stage, and 400 to the Adult stage. We found 317 shared lncRNAs between the Pre and Climax stages, 631 common lncRNAs between the Climax and Adult stages, 92 lncRNAs in both the Pre and Adult stages, and 1,148 lncRNAs in all stages.

### Co-expression network analysis and functional inference

To identify potential biological roles of these lncRNAs, we performed a Weighted Global Correlation Network Analysis (WGCNA) and obtained a total of 45,845 genes distributed across 122 modules (Supplementary Fig. 10). We filtered only those modules that included new putative lncRNAs to perform gene enrichment analysis in Gene Ontology. We obtained a total of 32 lncRNAs across 18 modules.

**Fig. 4.**
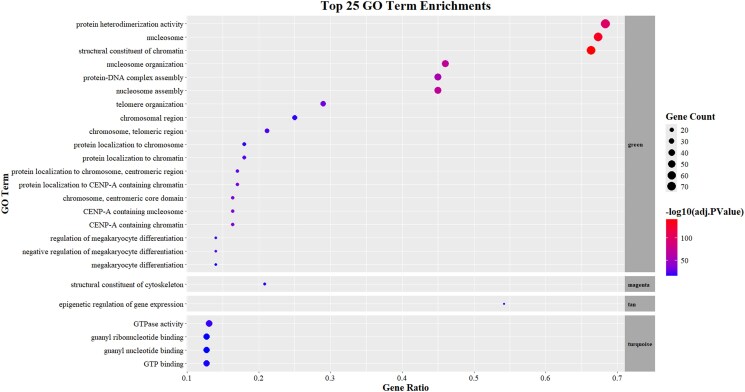
Gene enrichment analysis of lncRNA-containing co-expression modules top 25 enriched GO terms by module. We found enriched processes such as protein heterodimerization, structural organization and composition of the chromatin, organization of telomeres and chromosomes, epigenetic regulation, and GTPase activity.

We performed Gene Ontology enrichment analysis (Fig. 4) on the genes within each module. The green module was enriched for processes related to protein heterodimerization, protein-DNA complex assembly, nucleosome and telomere organization, and structural components of chromatin. The tan module showed enrichment for epigenetic regulation, while the turquoise module was associated with GTP binding.

## Discussion

We assembled de novo transcriptomes for 3 different life cycle stages of *D. arenicolor*. Our analysis included the detection of previously annotated lncRNAs, identification of novel putative lncRNAs, and coexpression network analyses to infer their potential biological roles. We identified putative de novo lncRNAs by eliminating sequences with coding potential, known protein domains, or identified as potentially coding in this species.

We quantified the size of both individual sample transcriptomes and the references we constructed per stage (Fig. 1). There is an apparent tendency for the Climax to have the smallest transcriptomes in both total genes and total transcripts, nevertheless, we found no significant differences between stages (Fig. 1, a and b). Similar sizes were reported for the brains of *Bombina pachypus* (Chiocchio et al. 2022), and *Xenopus andrei* (Pownall et al. 2018). Although our results suggest stable transcriptome sizes across stages, expanding sample sizes could reveal subtle differences across life cycle stages of *D. arenicolor*. To our knowledge, there are no reports of differences in the size of the transcriptome of multiple stages of the life cycle in anurans. Instead, there are reports of differentially expressed genes between said stages that determine sex development. Tang et al. (2021), the development of the hindlimb muscle (Shu et al. 2021), and even tail reabsorption (Wang et al. 2022) to name a few.

Assembly quality metrics, including contig size and N50 values, showed no significant differences among stages. We evaluated different statistics from the resulting Trinity assemblies and the reference transcriptomes (Fig. 1, c and d). Although there is an apparent difference between stages in contig size and N50, the results were not statistically significant; equivalent results were obtained previously (Zhao et al. 2014; Smirnov et al. 2022). While absolute N50 values may seem modest, this is expected in de novo transcriptomes from non-model organisms, which often lack high-quality genomic references (O’Neil and Emrich 2013), and may be limited by insert size during library construction due to the use of 150 bp read length (Hara et al. 2015).

When we evaluated the completeness of BUSCO genes against Eukaryota database (Table 1) we found almost full representation, compared to Vertebrata the representation levels were around 90%, finally compared against Tetrapoda all our datasets showed percentages above the 80% threshold. Considering that our transcriptomes come from a single tissue of a non-model organism constructed with a modest sample, we achieved robust assemblies. Comparable results were previously reported (Smirnov et al. 2022) against Tetrapoda, and the higher taxa reference (Ospina et al. 2021; Chiocchio et al. 2022; Libro et al. 2022).

Alignment to LNCipedia revealed high numbers of known lncRNAs in the Pre-metamorphic and Adult stages, and a lower number in the Climax stage (Fig. 2). Conversely, most novel lncRNAs were detected during the Climax stage (Fig. 3). The high number of unique transcripts associated with the Climax likely reflects the extensive morphological and physiological remodeling occurring during this critical transition period (Miyata and Ose 2012; Paul et al. 2022); such as an increased Wnt signaling activity (Qi et al. 2021), purine and pyrimidine metabolism, or apoptosis of skin cells, and differentiation of epidermis (Corrie et al. 2024). This pattern is consistent with findings in other metamorphosing species including *Sarcophaga peregrina* (Shang et al. 2023) *Drosophila* (Chen et al. 2016), *Ciona savignyi* (Wei and Dong 2018), *Bombyx mori* (Fu et al. 2022), and the frog *Microhyla fissipes* (Liu et al. 2023).

Common lncRNAs across stages could be involved in housekeeping roles such as gene regulation, regulation of biosynthetic processes, and the structure of the heterochromatin (Grammatikakis and Lal 2022; Herman et al. 2022). Alternatively, lncRNAs only detected in 1 stage could be involved in stage-specific relevant biological processes such as regulation of eye photoreceptor cell development in the Pre-metamorphic stage (Fig. 2b) and the inactivation of the X chromosome in the Climax stage (Fig. 2c), examples of this have been reported previously such as lncRNA involvement in the correct development of the eye photoreceptors in mouse (Chen et al. 2021; Zhang et al. 2024) and the inactivation of the X chromosome through XIST (Lu et al. 2020; Aguilar et al. 2022).

After eliminating transcripts with coding characteristics, we identified just over 4,800 putative novel lncRNAs (Fig. 3), a result consistent with findings in *Rana catesbeiana* (Hammond et al. 2017). In contrast, significantly higher numbers have been reported (around 50,000 lncRNAs) in *R. arenarum* (Ceschin et al. 2020), and approximately 80,000 lncRNAs in *Pelobates cultripes* (Liedtke et al. 2022). In contrast, we found significantly fewer transcripts; however, these studies were based on whole-genome assembly and whole-organism transcriptomes, respectively (Liedtke et al. 2019; Ceschin et al. 2020; Liedtke et al. 2022).

Notably, our results suggest that the majority of functionally relevant lncRNAs during this crucial developmental window remain uncharacterized, highlighting a key area for future research. Given that lncRNAs highly exhibit tissue-specific expression patterns (Jiang et al. 2016), expanding the range of sampled tissues or performing whole-genome sequencing would likely increase the number of detectable lncRNAs in *D. arenicolor*.

The co-expression modules where putative de novo lncRNAs are involved showed enrichment in relevant molecular functions of lncRNAs (Fig. 4), including the spatial organization of chromatin at multiple levels like nucleosomes, heterochromatin, telomeres, and entire chromosomes. LncRNAs are increasingly recognized as key modulators of chromatin architecture. For example, Suv39h1as, an antisense lncRNA, modulates its locus (in cis) regulating pluripotency in mouse embryonic Stem Cells (Bernard et al. 2022), while HOTAIR alters gene expression during epithelial–mesenchymal transition by sequestering a lysine demethylase (Jarroux et al. 2021). There have been reports of LncRNAs that can regulate telomeric homeostasis, namely TERRA, and alterations in its expression pattern can lead to genomic instability (Oliva-Rico et al. 2022) and even cancer (Xu et al. 2024).

The main limitation of our work was our use of human annotations to identify known lncRNAs and predict their functions, as well as the enriched GO terms of the co-expression modules. This limitation emerges given the absence of detailed lncRNA reference datasets for amphibians and the limited available information about lncRNAs in the nearest phylogenetically related species (*Xenopus*). As currently there is not much evidence to validate their roles in non-model species, these results should be further investigated to elucidate their function in Anurans. This is the first de novo identification of lncRNAs for *D. arenicolor*. We provide evidence that both known and novel lncRNAs are expressed during brain development and metamorphosis and may participate in a range of essential regulatory functions.

## Supplementary Material

jkaf283_Supplementary_Data

## Data Availability

Both raw sequence files (SRA) and transcriptomes of individual samples and the global reference were deposited at NCBI under BioProject ID PRJNA1295574. The code used for this paper and a comprehensive list of software and versions can be found in the GitHub repository: https://github.com/DarkHe007/LncRNAs-in-Dryophytes-arenicolor/tree/main Supplemental material available at G3 online.
