# Supplementary figures and images for: Brain transcriptome analysis reveals novel long non-coding RNAs in Dryophytes arenicolor (Canyon Treefrog)
Source: G3 (Bethesda). 2025 Nov 22;16(2):jkaf283. doi: 10.1093/g3journal/jkaf283 (PMC12869070; doi:10.1093/g3journal/jkaf283)

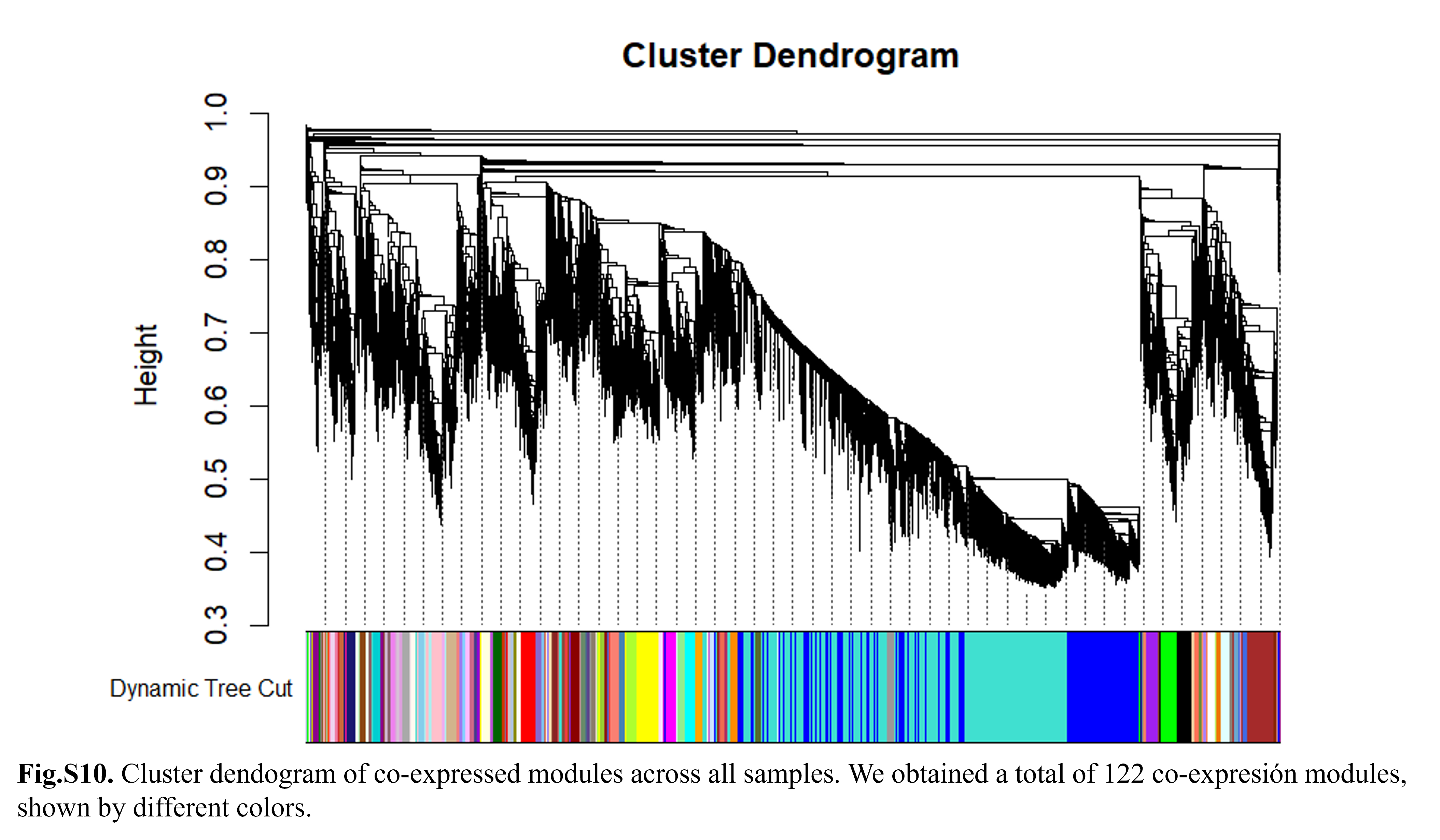

Supplement: jkaf283_Supplementary_Data [file jkaf283_supplementary_data.zip › Supplementary_Figure_10_G3-2025-406384.png]

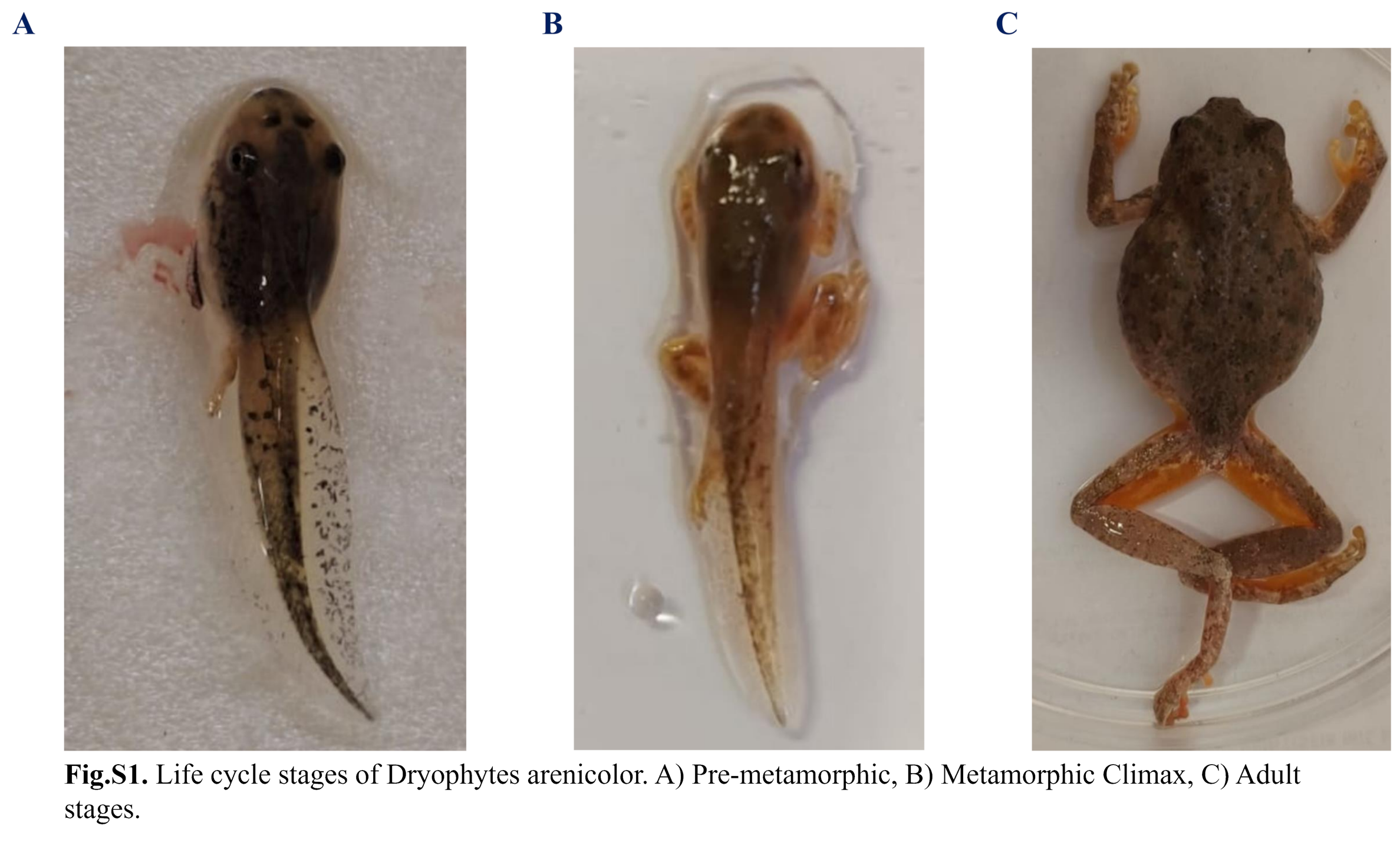

Supplement: jkaf283_Supplementary_Data [file jkaf283_supplementary_data.zip › Supplementary_Figure_1_G3-2025-406384.png]

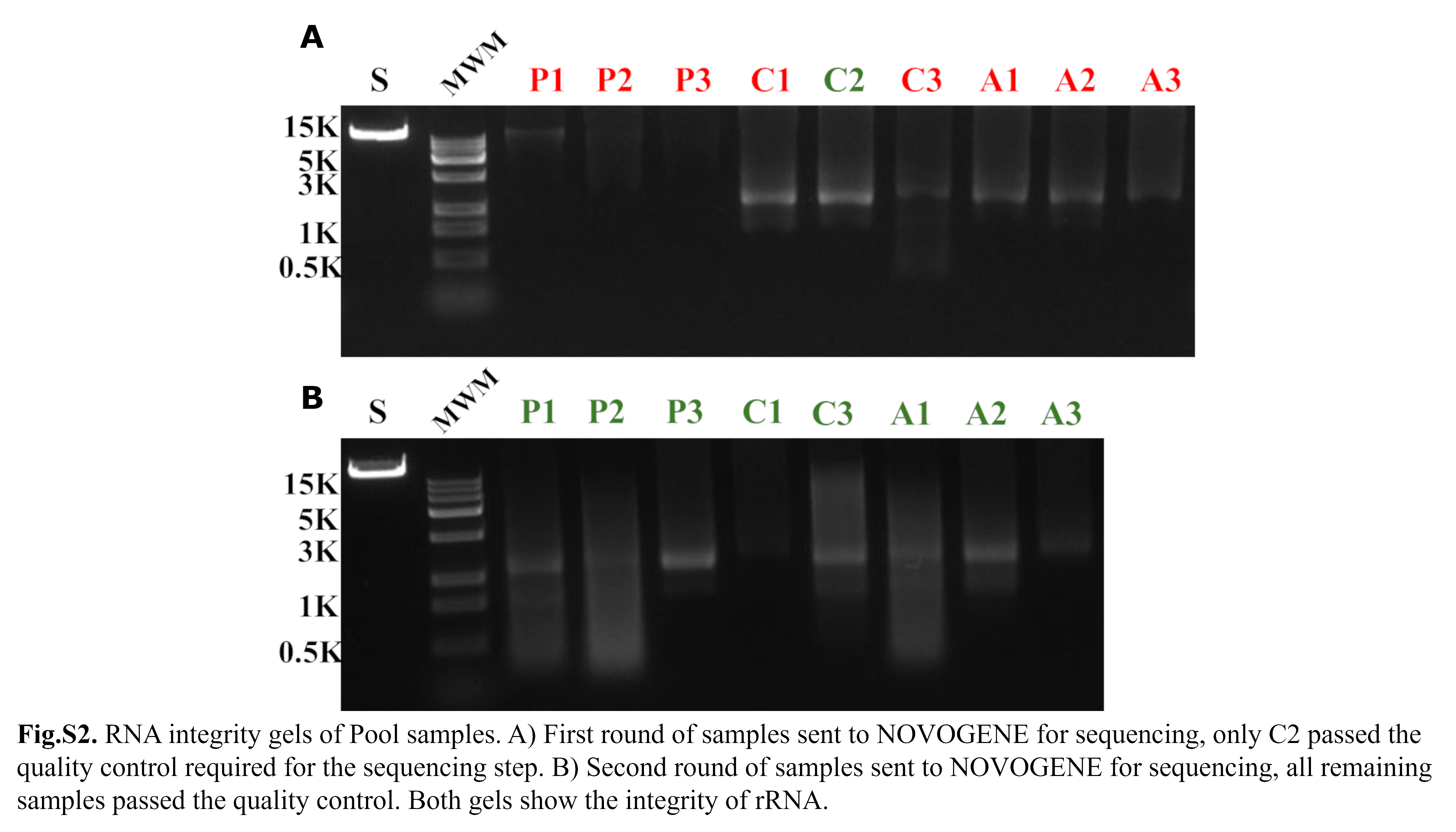

Supplement: jkaf283_Supplementary_Data [file jkaf283_supplementary_data.zip › Supplementary_Figure_2_G3-2025-406384.png]

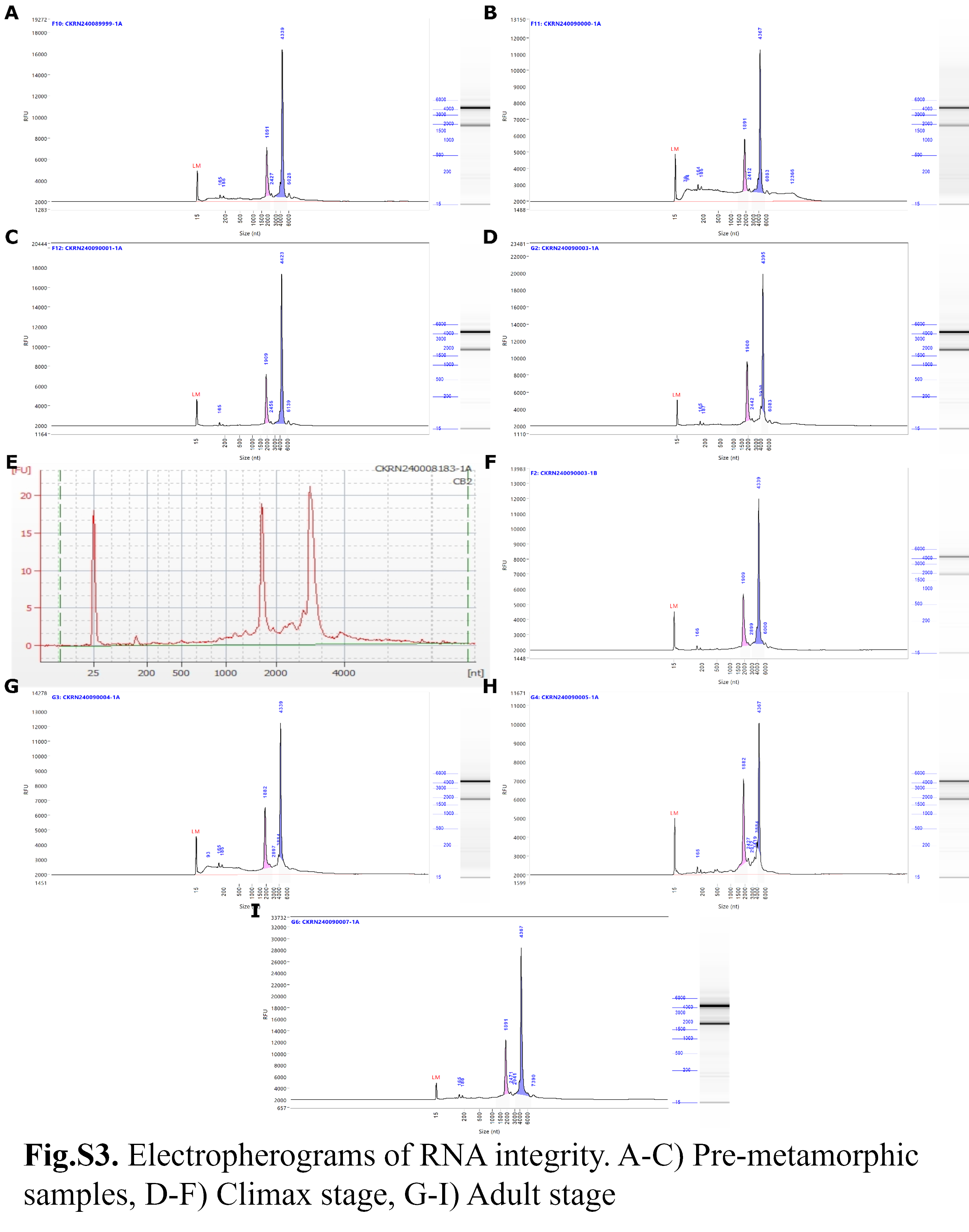

Supplement: jkaf283_Supplementary_Data [file jkaf283_supplementary_data.zip › Supplementary_Figure_3_G3-2025-406384.png]

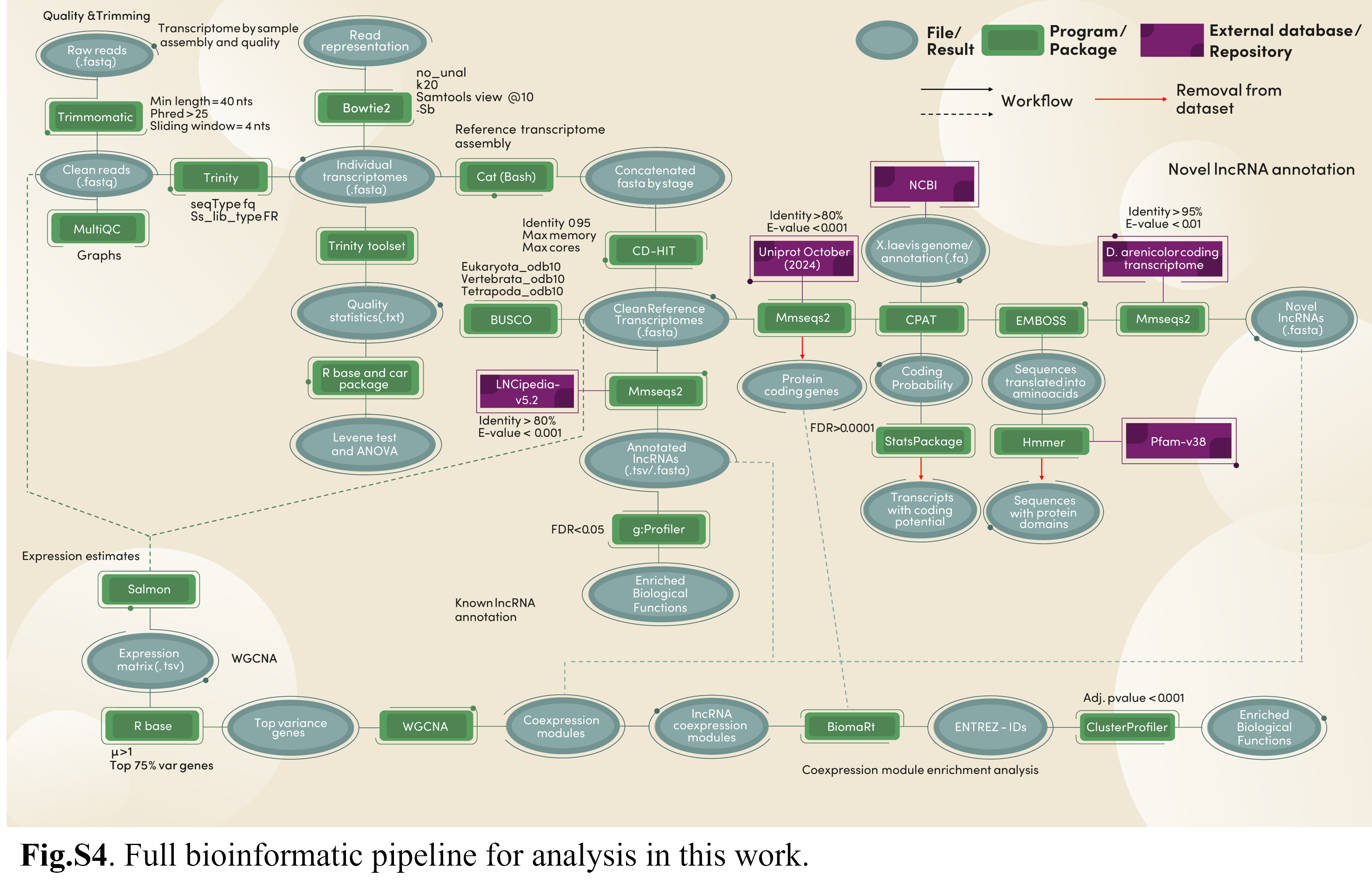

Supplement: jkaf283_Supplementary_Data [file jkaf283_supplementary_data.zip › Supplementary_Figure_4_G3-2025-406384.png]

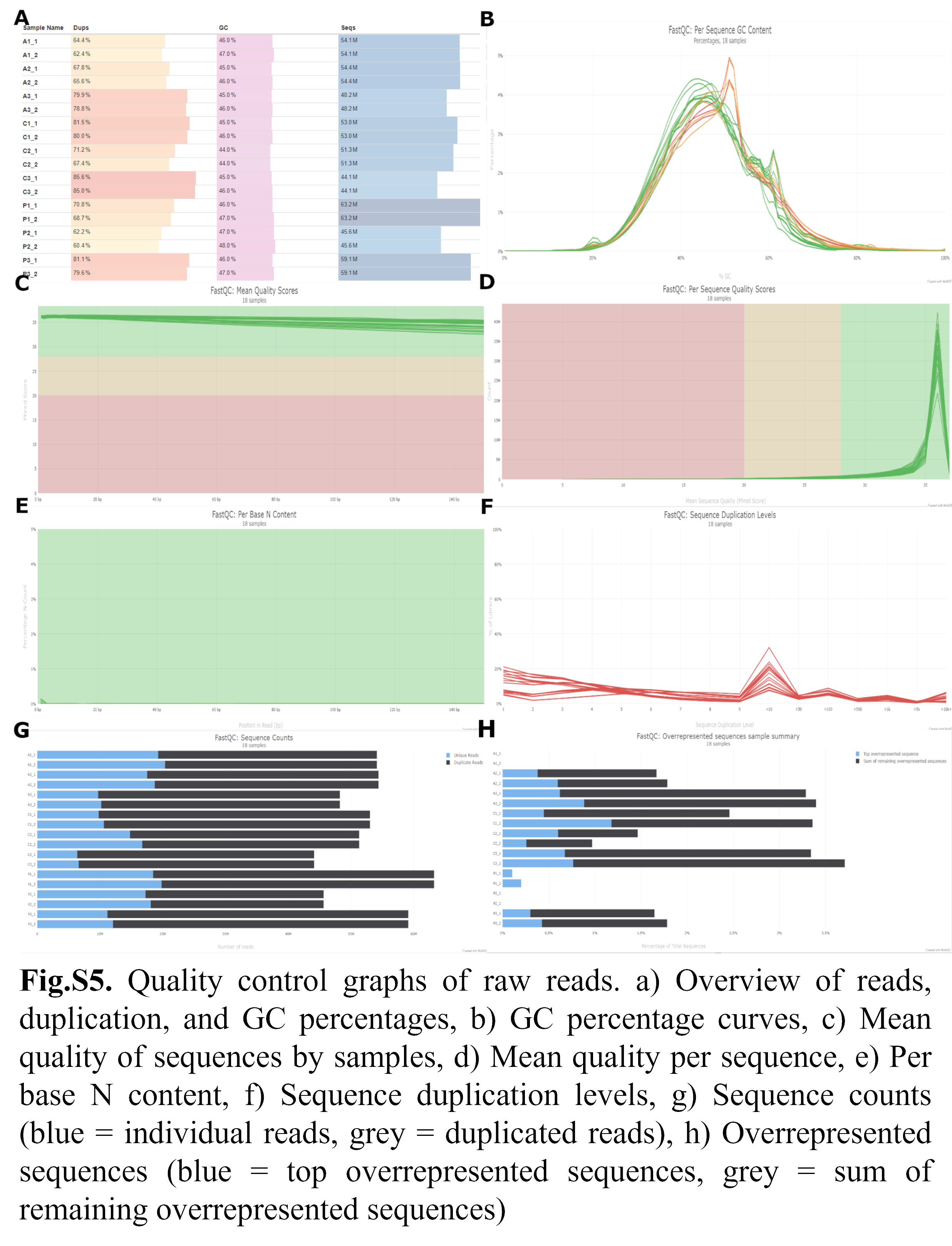

Supplement: jkaf283_Supplementary_Data [file jkaf283_supplementary_data.zip › Supplementary_Figure_5_G3-2025-406384.png]

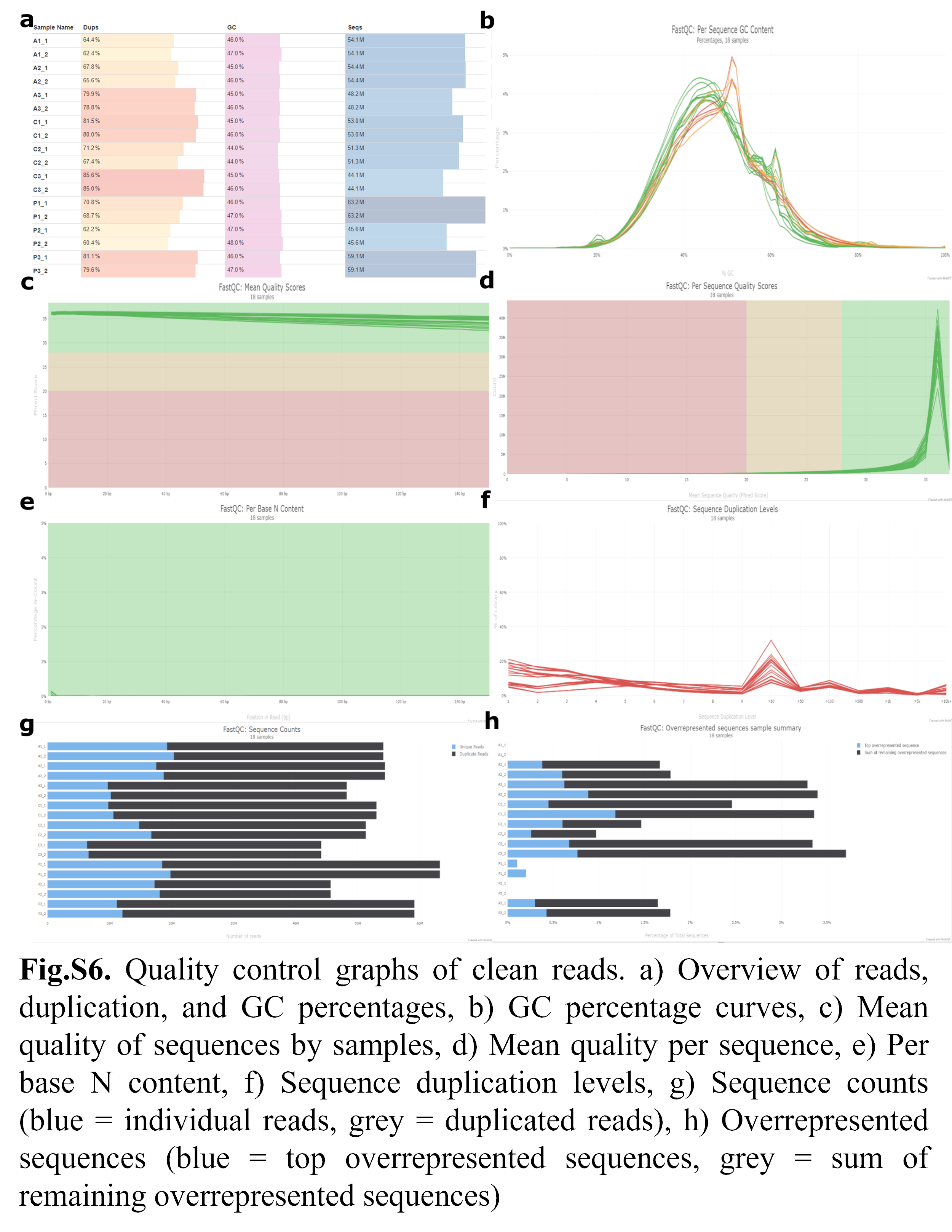

Supplement: jkaf283_Supplementary_Data [file jkaf283_supplementary_data.zip › Supplementary_Figure_6_G3-2025-406384.png]

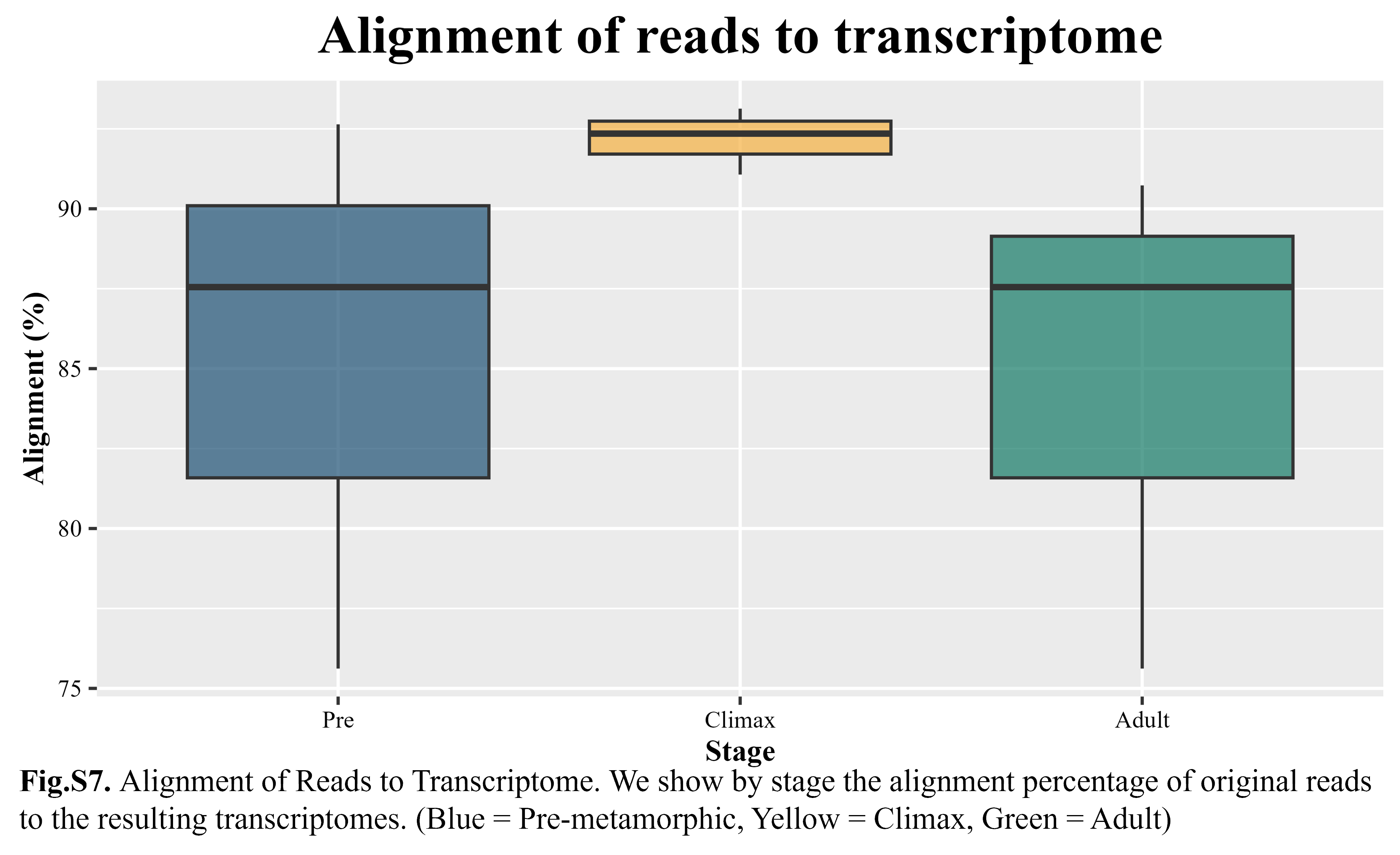

Supplement: jkaf283_Supplementary_Data [file jkaf283_supplementary_data.zip › Supplementary_Figure_7_G3-2025-406384.png]

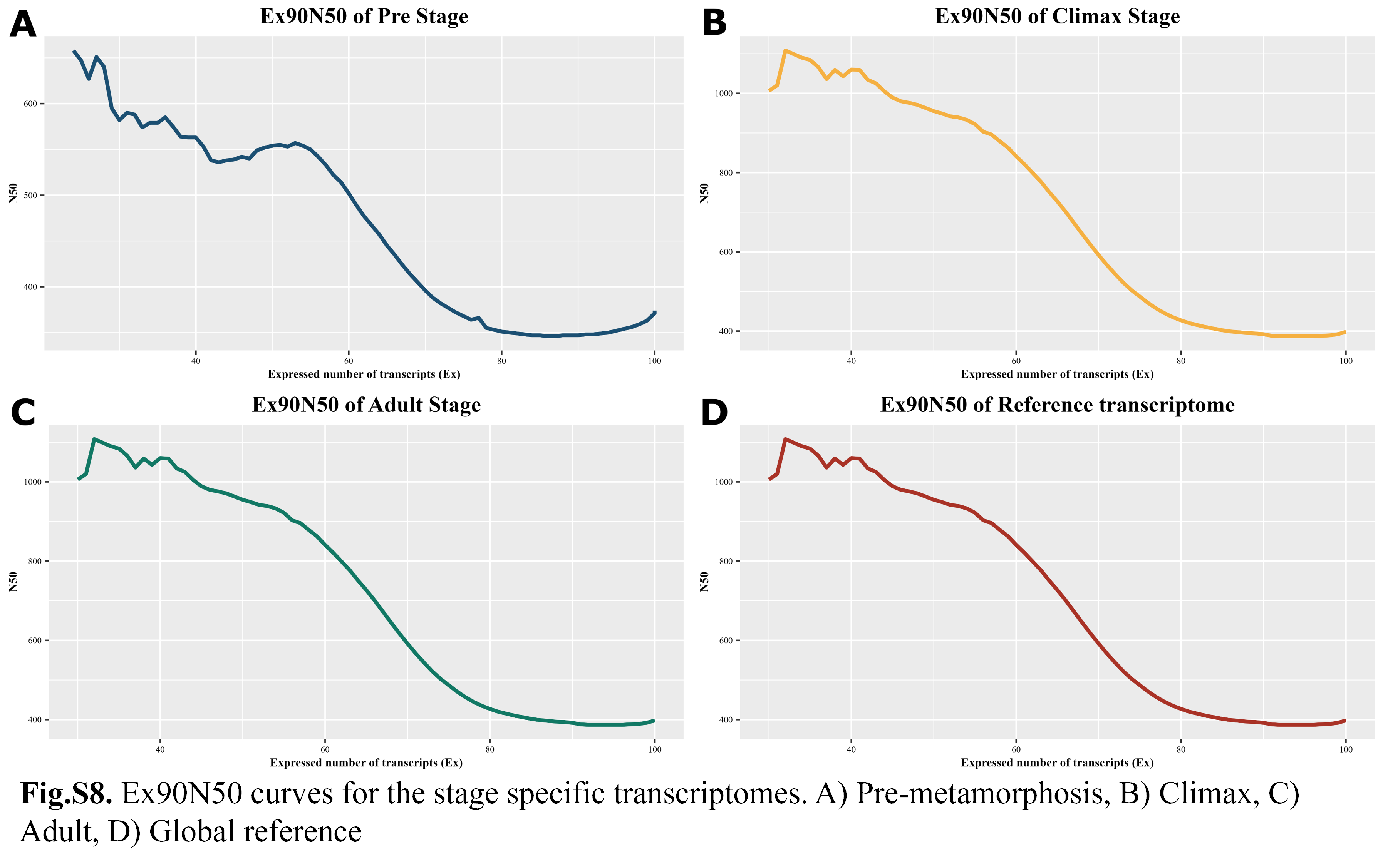

Supplement: jkaf283_Supplementary_Data [file jkaf283_supplementary_data.zip › Supplementary_Figure_8_G3-2025-406384.png]

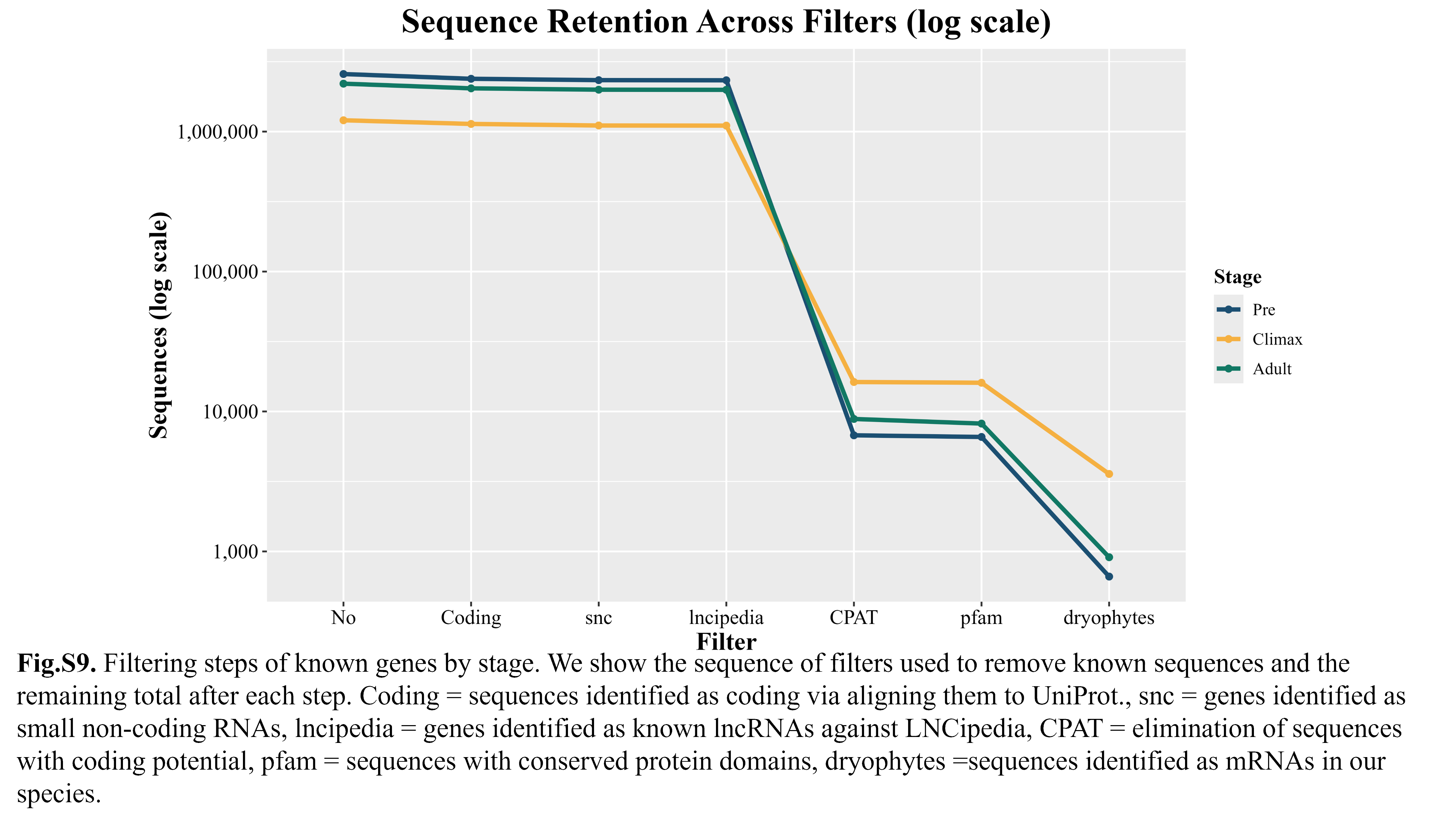

Supplement: jkaf283_Supplementary_Data [file jkaf283_supplementary_data.zip › Supplementary_Figure_9_G3-2025-406384.png]
